# A Superior Liquid Glue

**Published:** 1869-05

**Authors:** 


A Superior Liquid Glue.—A liquid glue, far superior to mucilage, may be made by dissolving glue in an equal quantity of strong hot vinegar, adding a fourth of alcohol and a little alum. This will keep any length of time when placed in closed bottles, and will glue together horn, wood, and mother of pearl.—*Scientific American.*

